# A broadband acoustic stimulus is more likely than a pure tone to elicit a startle reflex and prepared movements

**DOI:** 10.14814/phy2.12509

**Published:** 2015-08-26

**Authors:** Anthony N Carlsen

**Affiliations:** School of Human Kinetics, Faculty of Health Sciences, University of OttawaOttawa, Ontario, Canada

**Keywords:** Intensity, reaction time, reticular formation, startle, stimulus frequency

## Abstract

A loud acoustic stimulus that elicits a startle reflex has long been used to study the neurophysiology of cortical and subcortical neural circuits. More recent investigations have shown that startle can act as an early trigger for prepared actions, suggesting a brainstem role in the preparation and initiation of actions. However, in order to attribute any startle-triggered voluntary responses to activation in subcortical structures it is necessary to measure a startle-related activity in these structures. The current study investigated the most effective stimulus for eliciting a detectible startle reflex. While more intense stimuli are more likely to elicit a startle reflex, the current study examined whether broadband noise is more likely than a pure tone to produce a startle at various intensities above 100 dB. Participants performed a button release reaction time task in response to either a 1 kHz tone or a broadband noise pulse with intensities ranging from 82 to 124 dB. Reaction time and EMG from the wrist extensors and the sternocleidomastoid (SCM) were measured. Results showed that startle-related SCM EMG was elicited more frequently by broadband noise compared to pure tones. The higher proportion of startle reflexes observed in SCM was associated with a higher incidence of the voluntary task being triggered early. A higher incidence of startle following broadband noise is attributed to the activation of a larger proportion of the basilar membrane; thus, a lower intensity broadband noise stimulus may be used to elicit startle reflex at a similar rate as a higher intensity pure tone.

## Introduction

The startle reflex, a whole-body physiological response to a sudden and intense stimulus, has been employed in many studies as a probe to study basic neurophysiology and pathophysiology in humans (Landis et al. 1939). More recently, startle has also been used to probe voluntary response preparation by presenting a loud acoustic stimulus that is capable of eliciting a startle reflex in place of the go-signal in simple reaction time (RT) tasks (see Carlsen et al. 2012; Valls-Solé et al. 2008 for reviews). Findings have consistently shown that presenting a stimulus that elicits a startle also leads to a large facilitation of RT that is greater than can be explained by the increased stimulus intensity alone (Carlsen et al. 2007). It has been argued that startle trial RTs are so short (e.g., some premotor RTs <65 msec) that the normal cortical stimulus–response circuits must have been bypassed. These fast responses, termed “StartReact” responses, are thought to result from startle-related neural activation in subcortical structures interacting with voluntary response channels, leading to the prepared action being triggered involuntarily (Carlsen et al. 2012). Various studies have employed this StartReact effect to investigate the structures and processes involved in the preparation for various motor tasks from ballistic wrist extension (Valls-Solé et al. 1999) to gait initiation (Mackinnon et al. 2007) in both healthy controls and various motor disordered populations, such as patients with Parkinson’s disease (Carlsen et al. 2013) and stroke (Honeycutt and Perreault 2012).

In order to be able to confidently assert that any RT speeding following a startling acoustic stimulus (SAS) is the result of activation in subcortical areas related to the startle reflex, a reliable indication that a startle reflex actually occurred is required. While the eye-blink response is typically used to indicate a startle due to its consistency (Blumenthal et al. 2005), it may simply be too sensitive to loud acoustic stimuli to reliably predict sufficient activation of subcortical structures to elicit a StartReact response. A startle-related blink seen in the orbicularis oculi (OOc) is typically elicited in a large majority of trials by stimuli of only 100 dB (Blumenthal and Berg 1986; Brown et al. 1991). In contrast, although a startle-related burst of EMG is infrequently elicited in the sternocleidomastoid (SCM) with stimuli of less than 100 dB, the incidence of this response increases along with increasing acoustic stimulus intensity up to 124 dB (Carlsen et al. 2007). Importantly, it appears that in order to elicit a StartReact response (i.e., the early and involuntary release of the prepared action Carlsen et al. 2011, 2007, 2003), a *sufficiently strong* startle reflex must be elicited to also result in a burst of activity in the (presumably higher threshold) SCM (Carlsen et al. 2007). Observing an eye-blink response alone results in RTs that scale with stimulus intensity, and are no different than those produced by the well-known stimulus intensity effect (Kohfeld 1971). In contrast, when accompanied by SCM activation, RTs are significantly shorter than those produced when only OOc activity is present, and do not scale with intensity (Carlsen et al. 2007).

Thus, for studies using StartReact to probe motor preparatory-related activation of subcortical structures, it is advantageous to utilize an acoustic stimulus that results in the highest occurrence of SCM activation. It is well recognized that higher intensity stimuli lead to a greater likelihood of observing a startle reflex (even once the blink response has saturated), but it is prudent to consider limiting participant exposure to high-intensity acoustic stimuli. One potential way to reduce the intensity while maintaining the probability of causing a startle reflex is to employ broadband white noise as the acoustic stimulus instead of a pure tone (Blumenthal et al. 2005). For example, white noise has been shown to be more effective than a single frequency tone for eliciting the startle blink reflex at 95 and 100 dB (Blumenthal and Berg 1986), but it is currently unknown how stimulus frequency content may affect the incidence of observing an EMG response in SCM (as well as StartReact RT latencies) when used with the higher intensity stimuli that are typically used in StartReact studies (>115 dB). Therefore, the purpose of the current study was to investigate whether the frequency content of an acoustic stimulus would affect the proportion of trials where a SCM startle reflex and/or StartReact response was elicited at various intensities ranging from 100 to 124 dB. It was hypothesized that a broadband stimulus would result in a higher probability of both eliciting a startle and also triggering the prepared action (i.e., StartReact) compared to a pure tone at all intensities above control (82 dB).

## Experimental Procedures

### Participants

Sixteen neurologically healthy participants (6F, 10M; mean age = 24 years, SD = 6, range = 19–40 years) with no sensory or motor dysfunctions and with reported normal hearing volunteered to participate in the study. Three participants did not show a consistent startle reaction in SCM (see Data reduction and analysis section) and their data were excluded. Thus, analysis is based on data from 13 participants (5F, 8M; mean age = 23 years, SD = 6, range = 19–40 years). All participants gave written informed consent prior to taking part in the study. This study was approved by the Health Sciences and Science Research Ethics Board at the University of Ottawa (certificate H05-11-06) and conformed to the latest revision of the Declaration of Helsinki.

### Apparatus and task

Participants sat facing a 17-inch LCD computer monitor with their right arm resting palm down on a desk with the elbow flexed approximately 70°. The participant’s hand was resting on a microswitch such that the weight of the hand at rest was sufficient to close the switch. The experimental RT task required the participant to release the switch as quickly as possible following an auditory imperative go-signal using a rapid right wrist extension. Although the movement was untargeted, participants were instructed to use only a wrist movement to perform the task and to not lift the arm. A warning tone was followed by a variable foreperiod (2500–3500 msec, duration unknown to the participants), and finally by an acoustic go-stimulus which was either a 1 kHz tone or a broadband noise pulse 40 msec in duration. Instructions stated that the loudness of the stimulus would be variable, and emphasized fast reaction times in response to the sound.

The 40-msec go-signal was generated using a customized computer program written in LabView (National Instruments) and output using digital to analog hardware (PCI-6052E, National Instruments). The signal consisted of either a 1000 Hz sine wave or a broadband noise waveform (generated using a uniform white noise generation function in LabView – see Data S1) output at 22 kHz (output waveform power was similar from 1 Hz to 22 kHz). Each stimulus was amplified and presented via a loudspeaker (MG Electronics M58-H, frequency response 300 Hz–11 kHz) located directly behind the participant. Stimuli were calibrated to produce a peak impulse intensity of 82, 100, 108, 116, or 124 dB (A-weighted) using a data logging sound level meter (Cirrus Research Optimus, CR:162C) placed along with a Styrofoam head-form at a location corresponding to the position of the right ear of the participant during testing. For both broadband noise and tone stimuli, participants performed 32 RT trials with acoustic stimuli at 82 dB (control) and eight trials at each of the other four intensities (128 RT trials total). Presentation of stimulus type and intensity was pseudorandomized by the computer program whereby no two consecutive stimuli of 116 dB or above were presented. This practice ensured that the highest intensity stimuli were only presented in one of every four trials, which is similar to SAS presentation rates used previously (Carlsen et al. 2011). Nevertheless, even though the total proportion of trials with intensities higher than 82 dB was 0.5, it has been previously shown that when participants are preparing for a reaction task, startle response habituation is highly attenuated (Carlsen et al. 2003). Thus, any concern that a higher proportion of loud trials affecting the results was mitigated.

Movement onset RT was detected when the microswitch signal changed by more than 1 V following the stimulus and this was provided as feedback on the computer monitor after each trial. EMG data were collected from the muscle belly of the right extensor carpi radialis longus (ECR), as well as the left sternocleidomastiod (SCM) using bipolar, preamplified (gain = 10) surface electrodes (Delsys Bagnoli DE-2.1) connected to an external amplifier (Delsys Bagnoli-8). The recording sites were lightly scrubbed with a pumice gel and wiped using alcohol swabs in order to decrease electrical impedance. The electrodes were attached using double-sided adhesive strips whereby the sensor contacts were oriented such that the axis of the contact separation aligned with the direction of force generation of the muscle fibers. Thus, the sensor bars were each oriented perpendicular to the muscle fibers. A reference electrode (Q-Trace 5400, Covidien Inc.) was placed over the right lateral epicondyle. Raw bandpassed (20–450 Hz) EMG as well as the microswitch signal (to provide RT feedback) were digitally sampled at 1 kHz (National Instruments PCI-6052E via BNC-2090) for 3 sec, beginning 500 msec prior to the imperative stimulus, and stored for offline analysis.

### Data reduction and analysis

In order to confirm stimulus frequency content, noise and tone stimuli of 1 sec duration were used to analyze stimulus intensities in each of 10 frequency octave bins (31.5 Hz–16 kHz) calculated at 60 msec intervals using a data logging sound level meter (Cirrus Research Optimus, CR:162C). Means for each frequency bin were calculated from 10 of the steady-state intervals to provide a mean A-weighted sound pressure level (SPL) value in dB for each frequency octave for each of the stimulus intensities and types.

Surface EMG burst onsets in wrist extensors and SCM were identified using a thresholding method described previously (see Carlsen et al. 2011). In brief, the point at which the EMG first began a sustained rise above baseline levels was determined as the point at which the level of EMG activity in a rectified and filtered (25 Hz, dual pass, low pass second order elliptic filter) trace increased to more than 2 SD above baseline (mean of 100 msec of EMG activity preceding the go-signal). Onset was then verified by visually locating and manually adjusting the onset mark, if necessary, to the point at which the activity first increased. This method allows for correction of errors due to the strictness of the algorithm (see Carlsen et al. 2011; Hodges and Bui 1996 for details). Premotor RT was defined as EMG onset in the ECR muscle. To determine SCM startle incidence, trials were separated by whether or not an EMG burst was noted and marked in SCM in less than 100 msec following stimulus onset (see Carlsen et al. 2011 for details, Carlsen et al. 2007). A 100 msec cut-off criterion was chosen to limit the possibility of SCM activation due to voluntary contraction. Three of the original 16 participants did not show a reliable SCM response (<10% of total trials with stimuli 100 dB or greater), and thus these participants were excluded from the analysis. This proportion of “nonresponders” is similar to previous reports (Carlsen et al. 2004a). Premotor RT was then calculated for trials where a startle-related burst of EMG was observed in SCM (SCM+), and separately for trials where no SCM burst was observed (SCM−) (Carlsen et al. 2007).

### Statistical analyses

Dependent measures were analyzed using analysis of variance (ANOVA) procedures described below where appropriate to determine if differences existed between trials. Prior to analysis proportion data were subjected to an arcsine square root transform to correct for normality (Howell 2010). Greenhouse–Geisser corrected degrees of freedom were used if necessary to correct for any violations of sphericity. Partial eta squared (*η*_p_^2^) is reported to provide an estimate of effect size. Tukey’s HSD post hoc tests, as well as preplanned multiple comparisons using Holm–Bonferroni corrected Student’s paired *t*-tests were administered to decompose significant effects. Differences with a probability of less than 0.05 were considered to be significant.

## Results

### Stimulus characteristics

Differences in SPL at each octave were analyzed between broadband noise and tone stimuli using Holm–Bonferroni corrected Student’s *t*-tests. Analysis showed that for all intensities SPL was significantly greater (*P *<* *0.05) in all frequency bins above 250 Hz for broadband noise with the exception of the 1 kHz bin. SPL in the frequency bin centered about 1 kHz was significantly greater for the 1 kHz tone than for the broadband noise.

### Startle reflex

**Figure 1:**
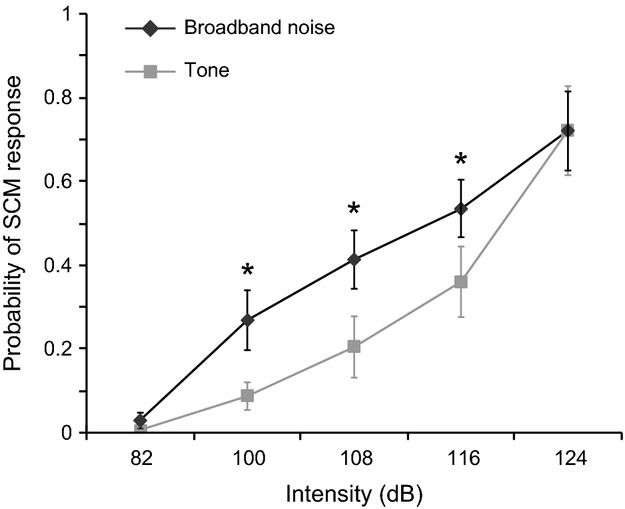
Probability of observing a startle response in sternocleidomastoid (SCM). Mean (SE) probability is shown across stimulus intensities for 1 kHz tone (gray) and broadband noise (black). *indicates significant difference between stimulus types at each intensity (*P *<* *0.05).

The proportion of trials where a startle-related SCM burst was observed for each stimulus type and intensity is shown in Figure1. Analysis of these data using a 2 (stimulus type: tone vs. noise) × 5 (dB level: 82, 100, 108, 116, 124 dB) repeated measures ANOVA revealed a main effect for stimulus type, *F*(1, 12) = 11.79, *P *=* *0.005, *η*_p_^2^ = 0.496, indicating that a startle response was elicited more frequently by the broadband noise stimuli. A main effect was also found for intensity, *F*(4, 48) = 40.77, *P *<* *0.001, *η*_p_^2^ = 0.773, indicating that a startle response was elicited more frequently with increasing intensity. A significant interaction between the factors was also found, *F*(4, 48) = 2.56, *P *=* *0.050, *η*_p_^2^ = 0.176. Holm–Bonferroni corrected Student’s *t*-tests comparing the percentage of trials in which a startle response was elicited by tone and noise at each intensity showed that startle activation was detected in a higher proportion of trials following broadband noise compared to the tone at 100, 108, and 116 dB (*P *<* *0.05); however, there were no differences between the stimuli at 82 or 124 dB. SCM onset latency was analyzed for stimulus intensities ≥100 dB using a 2 (stimulus) × 4 (intensity) repeated measures ANOVA. For some participants no startle responses were observed at certain intensities for one or both stimulus types; therefore, missing cells were filled using a linear regression-based multiple imputation procedure in SPSS (IBM Inc.) (max. filled cells per condition = 7/13, total in analysis = 19/104). No differences were observed in SCM onset latency between either the stimuli (*P *=* *0.584) or intensities (*P *=* *0.096), and there was no interaction between the variables (*P *=* *0.448).

### Voluntary response

**Figure 2:**
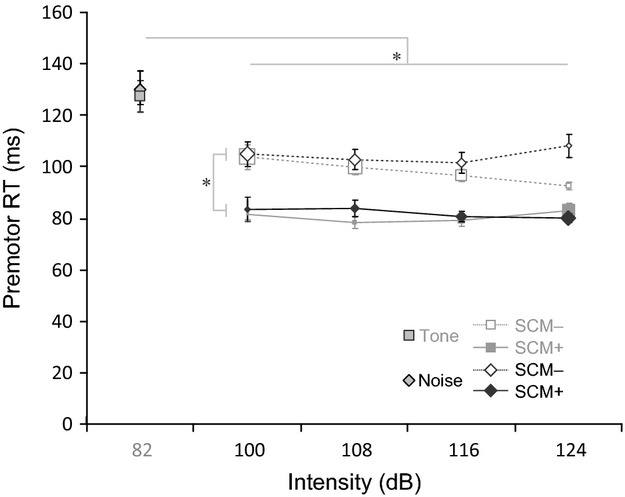
Premotor reaction time (RT) across intensities and stimulus types. Mean (SE) premotor RT is shown for 1 kHz tone (gray) and broadband noise (black), and whether or not a SCM startle response was (SCM+: solid lines) or was not (SCM−: dashed lines) observed, as a function of stimulus type and intensity. Note that each mean is made up of a different number of trials (see Results section for details), thus for intensities above control (82 dB) the *size* of the markers at each intensity represents the proportion of trials that makes up that mean. *indicates a significant (*P *<* *0.05) difference between factors.

In order to determine whether stimulus frequency content had a differential effect on RT as a function of stimulus intensity, premotor RT was compared between intensities and stimulus types using a 2 (stimulus) × 5 (intensity) repeated measures ANOVA (uncollapsed data are presented in Fig.2). This analysis revealed a significant main effect for stimulus intensity, *F*(4, 48) = 32.40, *P* < 0.001, *η*_p_^2^ = 0.730, but there was no main effect of stimulus type (*P* = 0.911) and no interaction between intensity and stimulus (*P* = 0.720). Tukey’s HSD post hoc tests showed that premotor RTs at all intensities above 82 dB were shorter than control (*P* < 0.01), but there were no differences between intensities above 82 dB.

As shown in Figure1, there was a difference in the proportion of trials that resulted in a startle response in SCM between the stimulus types. Thus, to determine whether RT was *differentially* affected by the presence of a SCM startle response, premotor RT was compared between the intensities of 100 dB and greater, stimulus types, and whether or not a SCM response observed (i.e., SCM+ vs. SCM−) using a 2 (stimulus) × 4 (intensity) × 2 (SCM presence) repeated measures ANOVA. Trials from the 82 dB stimuli were excluded from this analysis because there were too few trials where a SCM response was observed at the lowest intensity (nine trials total) to create a SCM+ mean. In addition, it should be noted that the number of trials that makes up each mean changed with stimulus intensity (illustrated as relative symbol size in Fig.2), such that more individual trials were used to calculate each SCM+ RT mean as intensity increased, whereas fewer trials made up the SCM- RT means at the higher intensities (see Fig.1). For some participants no SCM startle responses were observed at certain intensities (or *all* trials included a SCM startle) for one or both stimulus types; therefore, to perform the analysis, missing cells were filled using a linear regression-based multiple imputation procedure in SPSS (IBM Inc.) (max. filled cells per condition = 7/13, total in analysis = 32/208). Analysis revealed a significant main effect for SCM presence, *F*(1, 12) = 77.63, *P* < 0.001, *η*_p_^2^ = 0.866, indicating that SCM+ trials had significantly shorter premotor RTs than SCM− trials (Fig.2). In addition, there was a significant three-way interaction between the factors, *F*(3, 36) = 2.91, *P* = 0.048, *η*_p_^2^ = 0.195. Preplanned comparisons using Holm–Bonferroni corrected Student’s *t*-tests revealed that for both stimulus types the observed RT in SCM+ trials was significantly shorter than SCM− trials at each intensity (all *P* values <0.05), whereas no differences existed in RT between stimulus types when a SCM burst was (SCM+) or was not (SCM−) detected. Post hoc testing did not bear out the source of the three-way interaction. Looking at the partitioned data in Figure2, it appears that the three-way interaction might stem from a moderately faster premotor RT at 124 dB for tone stimuli compared to broadband noise when no SCM was present. However, because only this data point is representative of 28% of trials where the 124 dB tone was presented (i.e., a SCM response *was* noted on 72% of trials), it should be regarded with caution. There were no other significant main effects or interactions.

## Discussion

The present study was carried out to determine the effectiveness of a broadband noise stimulus versus a 1-kHz tone for eliciting startle response in SCM at intensities at or above 100 dB. Furthermore, it was of interest whether any stimulus-related differences would be evident in the early release of a prepared and intended response (StartReact). Results showed that the broadband noise stimulus led to a greater proportion of trials where a startle response was observed in SCM at all intensities above 82 dB except the highest (124 dB) compared to the pure tone (Fig.1). Previous studies have shown that broadband noise is more likely to result in a startle-related *blink* response in OOc than a pure tone at 95 and 100 dB (Blumenthal and Berg 1986); however, the present result is novel in that a similar effect was shown in SCM at higher intensities where OOc incidence tends to saturate. While the blink reflex has been commonly used to indicate the presence of a startle, evidence suggests that it is a lower threshold startle component compared to other muscles (such as SCM), and possibly subserved by a distinct pathway (Brown et al. 1991). While an eye-blink is evoked by a vast majority of trials with stimuli over 100 dB, SCM activity on the other hand, is elicited in only a low proportion of trials with stimuli below 100 dB, and increases in incidence with increasing stimulus intensity up to at least 124 dB (Carlsen et al. 2007).

The reticular startle reflex circuit receives output from the cochlear nucleus following intense acoustic stimuli. If this output is sufficiently strong, reticular structures including the giant neurons of the nucleus reticularis pontis caudalis (nRPC) produce the stereotyped pattern of contraction of the musculature (known as the classic startle reflex) (Yeomans and Frankland 1996). Since broadband noise was more likely to elicit a startle-related burst of EMG in the SCM, it is probable that the number of nRPC giant neurons recruited was greater for broadband noise compared to a pure tone stimulus. It is well known that broadband noise excites a larger proportion of the basilar membrane (Hudspeth 2000), thus the neural output in response to noise would be expected to be larger at a given intensity when a larger proportion of the basilar membrane was excited by the broadband stimulus (as compared to a pure tone). Moreover, the larger spectral bandwidth of noise stimuli would be expected to carry more overall signal power, resulting in stronger overall activation of the basilar membrane from a signal of lower amplitude. Indeed, analysis of the measured signal intensity showed a higher sound pressure level at all frequency octaves (except for 1000–2000 Hz, as expected) for the broadband noise, while overall peak impulse intensity (A-weighted) was not different between stimulus types. In neonatal infants, low-frequency tones (e.g., 125–250 Hz) have been shown to elicit startle reactions more often than higher tones at a similar intensity since lower frequencies excite a relatively larger proportion of the basilar membrane (Hutt et al. 1968). While these lower frequencies could not be explored in the current study due to equipment limitations, a larger and more intense activation of the reticular structures following broadband stimuli likely underlies the increased proportion of SCM startle reflex responses observed in the present study.

In recent years, a startle has been paired with reaction time tasks to investigate processes and structures underlying motor preparation (Valls-Solé et al. 1999; Carlsen et al. 2004b, 2013; Mackinnon et al. 2007; Maslovat et al. 2009, 2014; Carlsen and Mackinnon 2010; Honeycutt and Perreault 2012; Drummond et al. 2013). Yet as noted above, at the intensities used in these studies (generally >115 dB), the blink reflex incidence encounters a ceiling effect (Blumenthal and Berg 1986; Carlsen et al. 2007) – so measuring activity in SCM is critical to determining which trials can be considered to show strong activation in brainstem structures. The earliest explanations for the mechanisms underlying the speeding of voluntary responses by startle were first proposed by Valls-Solé et al. (1999), who argued that because the voluntary response was produced at such a short latency following the startling stimulus, normal cortical initiation processes must have been bypassed. Even for the fastest responses (some premotor RTs less than 65 msec) the time required for a stimulus to activate auditory cortex is approximately 35 msec (Erwin and Buchwald 1986), while the time required for nerve conduction from primary motor cortex to the limbs is 20–30 msec (Rothwell 1997), leaving almost no time for cortico-cortical transmission and generation of the efferent volley. Thus, two possibilities were proposed to account for the low absolute latencies at which the startle-driven responses occurred. Because the voluntary responses were driven at a similar latency to startle reaction itself, it has been suggested that either (1) sufficient details of the response are “stored” in brainstem structures that are also responsible for the startle reaction (e.g., reticular formation) (Valls-Solé et al. 1999; Carlsen et al. 2004a); or (2) a cortically stored program is triggered by activation related to the startle via an alternate fast conducting pathway (e.g., reticulo-thalamo-cortical) (Alibiglou and Mackinnon 2012; Carlsen et al. 2012). It is important to keep in mind that both of the above explanations rely on the notion of activity in reticular formation acting to trigger the prepared response at a short latency. That is, the StartReact effect has been interpreted to reflect a subcortically mediated release of a prepared motor program.

In order to argue that the StartReact speeding following a loud acoustic stimulus is the result of activation of brainstem structures related to the startle response, it follows that some evidence of this activation is required. As such, in order to maximize these trials it would be advantageous to utilize a broadband noise stimulus that results in a higher proportion of trials exhibiting SCM responses as shown in the present study. However, it has also been argued that detecting the presence of a startle response in SCM is not *necessary* to result in StartReact response triggering (Marinovic et al. 2013). This reasoning is based on one study that showed that StartReact could be dissociated from the startle reflex in some cases, as prepulse inhibition of the startle reflex in SCM does not affect the speeding of voluntary responses (Maslovat et al. 2012). However, outside of this scope it has been demonstrated repeatedly, including in the present study (Fig.2), that RTs in trials where SCM activity is detected are different than those where no SCM is detected (Carlsen et al. 2007; Honeycutt and Perreault 2012; Honeycutt et al. 2013; Tresch et al. 2014; Maslovat et al. 2015). Nevertheless, some studies have attributed experimental results following the presentation of a loud acoustic stimulus to brainstem involvement *without* any indication of startle (Marinovic et al. 2014; Nonnekes et al. 2014). This practice assumes a similar mechanism was employed – an assumption that may not always be true. That is, without the measurement of brainstem activity (e.g., using the startle reaction), there is no way to know that the presentation of a loud stimulus had any effect on brainstem structures. As noted in the Introduction section, it is well known that more intense stimuli lead to faster RTs (Kohfeld 1971), but this cortically mediated pathway is not thought to involve any brainstem structures (Lakhani et al. 2012). Therefore, it is argued that without a separate indication that participants were startled (barring some other measure of brainstem activity), it is difficult to claim that the speeded response following a loud acoustic stimulus was due activity in brainstem structures.

Here, it is argued that an observed burst of SCM EMG constitutes sufficient indication of brainstem activation as RTs were significantly shortened when a burst of activity is SCM was detected (indicating activation in reticular structures), compared to when it was not. Furthermore, this effect occurred for both stimulus types whereby on average, RTs were shortened to a similar absolute latency (∼85 msec) when SCM activity was detected, irrespective of stimulus intensity. This is particularly striking when it is noted that the small proportion of trials that exhibited a burst of activity in SCM (Fig.1) following a 100 dB stimulus (noise = 27%; tone = 9%) show similar RTs (Fig.2) to the high proportion of trials at 124 dB with SCM (noise = 72%; tone = 72%). In addition, the low proportion of trials with no SCM activity following the 124 dB stimuli show less RT speeding, and are comparable to the RTs in the large proportion of trials with no SCM activity at 100 dB. Again, this strongly suggests that the StartReact effect is not simply stimulus driven. Even for different stimulus types and intensities, RTs were significantly shorter when a SCM response was observed. Although a smaller (yet nevertheless significant) difference in RT was observed between SCM+ and SCM− trials following the 124 dB tone stimulus, the number of SCM− trials contributing to this mean is comparatively small (represented as size of symbols in Fig.2). Although it is possible that some of these SCM− trials were indeed triggered by startle, but did not exhibit sufficient observable SCM activity, it is nevertheless suggested that these trials be separated for analysis in the interest of a conservative definition of StartReact. Finally, while it would be expected that an eye-blink response would be apparent on the vast majority of the trials with stimuli of 100 dB or more (Blumenthal and Berg 1986), the RT distributions were reliably differentiated when a SCM response was observed, and more of these trials occurred when broadband noise was used.

In summary, these results show that broadband noise stimuli led to a higher proportion of trials where a startle reflex was elicited in humans, up to 124 dB where it appeared to reach a ceiling (Fig.1). Furthermore, when a startle reflex was evoked in SCM the amount of RT facilitation that resulted was similar irrespective of the frequency content of the stimulus (Fig.2). Thus, at most of the tested intensities a StartReact response was also observed in a higher proportion of trials where broadband noise was used. To avoid damage to the hearing apparatus (NIOSH, 1998), prolonged exposure to high-intensity stimuli, (e.g., the number of SAS exposures) is often limited. By using lower intensity broadband noise instead of pure tone stimuli, experiments employing a SAS may be able to decrease the noise exposure for participants while achieving a similar number of startle responses.

## Supporting Information

**Data S1.** Supplemental Excel data are provided that contribute to the reported results.
