# Supplementary material for: A broadband acoustic stimulus is more likely than a pure tone to elicit a startle reflex and prepared movements
Source: Physiol Rep. 2015 Aug 26;3(8):e12509. doi: 10.14814/phy2.12509 (PMC4562592; doi:10.14814/phy2.12509)
Supplement: Supplementary file 2 [file phy20003-e12509-sd2.docx]

Supplemental Excel data are provided that contribute to the reported results. These include individual trial data for all participants, individual participant means by condition, as well as grand means. See Excel file for full annotation and notes. Pages described below.

Notes: information regarding the data provided in the Excel file.

Data: Full set of trial by trial data for all dependent measures for all participants.

S01-S13: Individual participant data for each dependent measure trial by trial. Means by condition are found at the bottom of each page.

Means: Grand means, SD, and SE for each dependent measure compiled from individual participant means.

SCM+-RT Imputed: Data used to impute missing RT values for SCM+ means and SCM- means.

SCM Onset Imputed: Data used to impute missing values for SCM onset.

Stimulus Intensities: Peak stimulus intensity measured at each of ten octaves for both stimulus types and for all intensities.

Noise Waveform: One second long noise file used during data collection. 22,000 data points ranging from -1 to +1.
